# A highly sensitive stem-loop RT-qPCR method to study siRNA intracellular pharmacokinetics and pharmacodynamics

**DOI:** 10.1093/biomethods/bpae029

**Published:** 2024-05-06

**Authors:** Lin Chen, Caroline Bosmajian, Sukyung Woo

**Affiliations:** Department of Pharmaceutical Sciences, School of Pharmacy and Pharmaceutical Sciences, University at Buffalo, State University of New York, Buffalo, NY 14214, United States; Department of Pharmaceutical Sciences, School of Pharmacy and Pharmaceutical Sciences, University at Buffalo, State University of New York, Buffalo, NY 14214, United States; Department of Pharmaceutical Sciences, School of Pharmacy and Pharmaceutical Sciences, University at Buffalo, State University of New York, Buffalo, NY 14214, United States

**Keywords:** siRNA, stem-loop RT-qPCR, RISC loading, intracellular pharmacokinetics

## Abstract

Small interfering RNA (siRNA) is a powerful tool for sequence-specific silencing of disease-related genes. In this study, we established and validated a stem-loop reverse transcription-quantitative polymerase chain reaction (RT-qPCR) method applicable for both chemically unmodified and modified siRNA, aiming to elucidate mechanistic intracellular pharmacokinetic and pharmacodynamic (PK/PD) properties of siRNA. We conducted a comprehensive evaluation of factors affecting intracellular siRNA quantification. Our study revealed that immobilization-based siRNA extraction introduced high variation, making it unsuitable for absolute quantification. Conversely, direct cell lysis followed by stem-loop RT-qPCR demonstrated excellent reproducibility, with a quantification range from 0.0002 to 20 femtomole (fmole) for unmodified siRNA and 0.02 to 20 fmole for modified siRNA. The design of a 6-bp overlapping RT primer facilitated the distinction of full-length antisense from its 3′-metabolites, and pre-annealing of antisense to RT primer enhanced sensitivity and reproducibility. Differences in siRNA loss during storage and sample processing were noted among microcentrifuge tubes from various manufacturers. Endogenous miR-16 served as a reference for normalizing cytoplasmic siRNA, while protein concentration post-immunoprecipitation lysis was used to normalize RNA-induced silencing complex (RISC)-loaded siRNA levels. This method successfully enabled a detailed characterization of the time profiles of cytoplasmic and RISC-loaded siRNA, advancing the *in vitro–in vivo* translation of siRNA therapeutics.

## Introduction

RNA interference (RNAi) is a natural biologic process that regulates gene expression at the post-transcriptional level [1]. By harnessing this intrinsic mechanism, RNAi-based therapeutics, theoretically capable of silencing any disease-related genes in a sequence-specific manner, are revolutionizing drug development for the treatment of diverse diseases [2–4]. Small interfering RNA (siRNA) is typically a double-stranded RNA with ∼20–30 nucleotides (nt) in length. After effective cellular delivery, cytoplasmic-free siRNA loads into the RNA-induced silencing complex (RISC) to mediate the cleavage of specific target mRNA [5]. Since the Food and Drug Administration (FDA) approved the first siRNA drug, Patisiran (Onpattro^®^; Alnylam), in 2018, a total of six siRNAs have been approved to date [6, 7].

To achieve the broad clinical application of siRNA therapeutics, extensive efforts have been made in the past two decades, especially in overcoming instability and delivery. These efforts include, but not limited to, the design of Dicer-substrate siRNA (DsiRNA) to enhance the gene silencing potency, extensive chemical modifications in ribose and backbone to increase siRNA enzymic stability, the application of lipid-based vehicles to encapsulate siRNA, and the development of conjugates, such as N-acetylgalactosamine (GalNAc), to facilitate siRNA delivery to target sites [4, 8–11].

Compared to small molecules or protein drugs, siRNA therapeutics exhibit a complex pharmacokinetics (PK) and pharmacodynamic (PD) relationship. The incorporation of cellularly delivered siRNA into RISC is a key bioactivation step for effective target gene silencing. Plasma PK does not directly correlate with the PD effect, posting a continuing challenge for the *in vitro* and *in vivo* translation of siRNA therapeutics [6]. Studies on the PK/PD properties of GalNAc-conjugated siRNAs have revealed that the plasma and liver dispositions across species are highly predictable, and the PD profiles correspond directly with the exposure of RISC-loaded siRNA [12–14]. To enhance the in *vitro–in vivo* correlation of siRNA therapeutics, further insights into intracellular disposition of siRNA, particularly the PK of RISC-loaded siRNA, is a critical need.

To better understand intracellular siRNA disposition, quantification methods with both desirable sensitivity and specificity are essential. While techniques such as mass spectrometry, hybridization-ELISA, and photodiode array have been proven useful for siRNA quantification, their sensitivity at the nanogram per milliliter range cannot meet the requirements for the trace amount quantification of intracellular siRNA [15]. In contrast, the reverse transcription-quantitative polymerase chain reaction (RT-qPCR) has been reported to show extremely high sensitivity for siRNA quantification, providing a more cost-effective and higher throughput way [16, 17].

To apply the RT-qPCR method for the accurate characterization of intracellular siRNA disposition, the reproducibility, and robustness are still the concerns. In this study, we comprehensively assessed the potential impacts of siRNA extraction methods, primer design, siRNA sample pre-processing, normalization references, and microcentrifuge tube adsorption on quantification sensitivity, specificity, and reproducibility. This study describes the development and validation of a direct cell lysis coupled with stem-loop RT-qPCR method for characterizing the intracellular disposition of both chemically unmodified and modified siRNA. The developed method was successfully applied to depict the time profiles of cytoplasmic and RISC-loaded siRNA. The stem-loop RT-qPCR method enhances our understanding of siRNA’s unique PK/PD properties, facilitating *in vitro–in vivo* translation and advancing the development of siRNA therapeutics.

## Materials and methods

### Cell culture

The human breast cancer cell line BT474 and liver cancer cell line Hep3B were purchased from the American Type Culture Collection (ATCC; Manassas, VA, USA). BT474 cells were grown in Dulbecco’s Modified Eagle’s Medium (DMEM) supplemented with 20% fetal bovine serum (FBS), 100 U/ml penicillin and 100 µg/ml streptomycin. Hep3B cells were cultured in Minimum Essential Medium (MEM), supplemented with 10% FBS, 100 U/ml penicillin, 100 µg/ml streptomycin and non-essential amino acids (1:100 dilution of 100X MEM NEAA). Cells were maintained in incubator at 37°C, 5% CO_2_, and 95% humidity.

### siRNA

Chemically unmodified DsiRNAs against human hypoxanthine phosphoribosyltransferase 1 (DsiHPRT1), peptidyl-prolyl cis–trans isomerase B (DsiPPIB), and glyceraldehyde 3-phosphate dehydrogenase (DsiGAPDH), along with negative control siRNA and reference microRNA miR-16, were purchased from Integrated DNA Technologies (IDT, Coralville, IA, USA). siRNA against proprotein convertase subtilisin/kexin type 9 (siPCSK9) with chemical modification and GalNAc-conjugated siPCSK9, sharing the same sequence, were purchased from MedChem Express (Monmouth Junction, NJ, USA). The sequences of these siRNAs are listed in Supplemental Table S1. siPCSK9 contains modifications at 2′-position of the ribose ring to a 2′-F or a 2′-OMe throughout the sequence and terminal phosphorothioate bonds to replace phosphodiester bonds to enhance stability against exonucleases. The tri-GalNAc moiety of siPCSK9 is conjugated to the sense strand at the 3′-end. Lyophilized siRNA and GalNAc-siRNA conjugate were reconstituted in IDT Duplex Buffer to a concentration of 100 µM, then further diluted to the desired concentrations and stored at −20°C.

### siRNA transfection and gene knockdown

Each well of the six-well plate was seeded with 250,000 BT474 and 200,000 Hep3B cells, allowing them to attach overnight. Lipofectamine RNAiMAX (Invitrogen) was used for the delivery of DsiHPRT1 and siPCSK9 into BT474 and Hep3B, respectively. For BT474, after replacing the full medium with Opti-MEM^™^ reduced serum medium (Gibco) on the day of transfection, cells were exposed to RNAiMAX-encapsulated negative control siRNA (1 nM) and DsiHPRT1 at final concentrations of 0.03, 0.3, and 1 nM for 24 h. Specifically, the final transfection mixture consisted of 1 μl of Lipofectamine RNAiMAX per 600 μl of Opti-MEM. Afterward, cells were washed with PBS and full medium was added for continued culture. At specific time points between 3 h and 17 days since the initiation of transfection, cell samples were harvested by trypsinization and centrifuged at 180*g* for 5 min. Cell pellets were then resuspended in PBS and counted using the Countess^™^ 3 automated cell counter (Invitrogen). Samples with a specific cell number were pelleted again at 2000*g* for 4 min and stored at −80°C until RNA extraction for quantifying siRNA cytoplasmic and RISC-loaded amounts, as well as mRNA levels. The same procedure was used for the transfection of 0.5 nM siPCSK9 in Hep3B. High asialoglycoprotein receptor (ASGPR) expression in Hep3B allows direct GalNAc-siPCSK9 delivery [18]. Hep3B cells were incubated with Opti-MEM^™^ reduced serum medium containing 100 nM GalNAc-siPCSK9 for 72 h, and samples were collected as described above.

### Immobilization-based siRNA extraction plus complementary DNA synthesis by poly(A) extension

One commonly adopted approach for siRNA extraction involves the acid-phenol: chloroform extraction followed by immobilization of RNA on glass-fiber filter. This column extraction method has been widely used for siRNA extraction [17]. Furthermore, another siRNA reverse transcription (RT) method involves the utilization of poly(A) polymerase to append a polyadenylate tail to the siRNA antisense strand, and the utilization of a poly(T) adapter as a reverse transcription primer. This method effectively enhances the complementary DNA (cDNA) length [19, 20]. The integration of this siRNA extraction method with a poly(A)-based RT-qPCR approach was also assessed for DsiHPRT1 quantification.

As previously detailed [17], mirVana^™^ PARIS^™^ RNA and Native Protein Purification Kit (Thermo Fisher) were employed for siRNA extraction. The extracted siRNA was then utilized as a template for cDNA synthesis, performed using the qScript^™^ microRNA cDNA Synthesis Kit (Quanta Biosciences). For the qPCR reaction setup, a master-mix consisting of 10 μl PerfeCTa^®^ SYBR^®^ Green SuperMix (Quanta Biosciences), 0.4 μl PerfeCTa^®^ Universal PCR Primer, 0.4 μl DsiHPRT/miR-16 forward primer (sequence: 5′-GCGCTGGTCATTACAATAGCTCTT-3′, 5′-TAGCAGCACGTAAATATTGGCG-3′), and 4.2 μl nuclease-free water was mixed with 5 μl of cDNA template. The thermal cycling program was set as follows: an initial 3 min denaturation at 95°C, followed by 40 cycles of 15 s at 95°C and 1 min at 60°C.

### Simple cell lysis for cytoplasmic siRNA extraction

The cell lysate was used directly for siRNA extraction from cell pellets, following a protocol similar to microRNA quantification [16, 21]. To isolate and stabilize cytoplasmic siRNA, approximately 20,000 cells were lysed using the iScript RT-qPCR sample preparation reagent (called “iScript SPR” hereafter; Bio-Rad Laboratories). Briefly, cell pellets (approximately 10 μl with residual PBS) were mixed with 100 μl of the iScript SPR and vortexed for 30 s at a medium setting. After centrifugation at 15,000*g* for 2 min, the resulting supernatant (approximately 70 μl) was carefully transferred to a new Eppendorf tube and stored at −80°C until the RT process.

For the preparation of spiked standard and quality control (QC) samples, 10 μl of serial stock solutions of siRNA were added to 100 μl of the iScript SPR. The subsequent steps followed the earlier procedures.

### Stem-loop RT-qPCR for siRNA quantification

**Figure 1.**
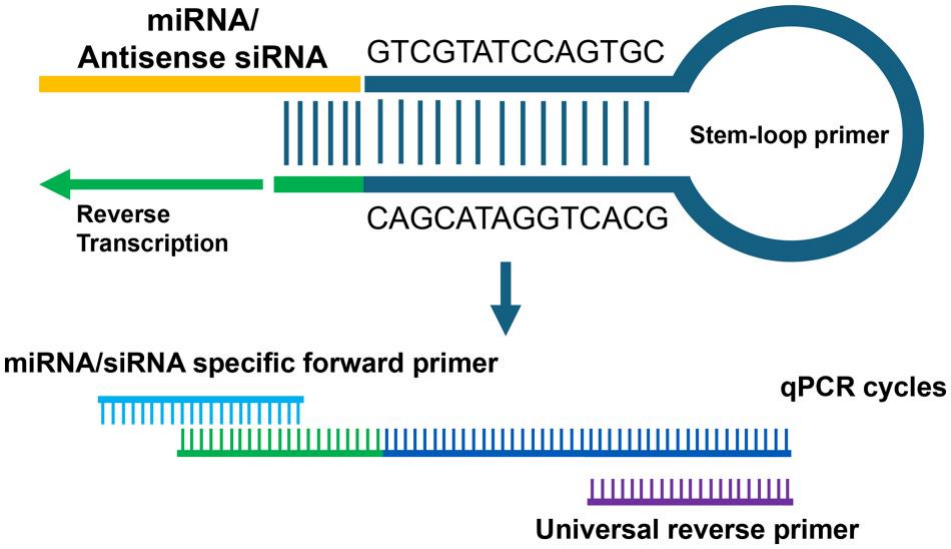
Schematic representation of the stem-loop RT-qPCR method.

Due to the small size of siRNA, a sequence-specific RT primer with a stem-loop structure was used to extend the length of the synthesized cDNA [16, 22]. The principle of stem-loop RT-qPCR is presented in Fig. 1. Apart from the universal sequence (5′-GTC GTA TCC AGT GCA GGG TCC GAG GTA TTC GCA CTG GAT ACG AC-3′) responsible for the formation of the stem-loop structure, the RT primer contains an additional 6-nt sequence at its 3′-terminus. This extra sequence is designed to perfectly complement the 3′-terminus of the antisense strand of the target siRNA. The sequences of stem-loop RT primers for different siRNAs are listed in Supplemental Table S2. The endogenous microRNA miR-16 was selected as the reference gene to normalize cell numbers because of its relatively abundant and constant expression in various cell lines [23, 24].

The TaqMan MicroRNA Reverse Transcription Kit (Thermo Fisher) was used for reverse transcription. Instead of simply mixing siRNA sample, RT primer and the other RT reagents and directly introducing to RT reaction, an additional pre-denaturation and annealing process was first applied between siRNA sample and RT primer. Briefly, 2 μl of spiked samples or cell lysate, obtained as described above, was mixed with a 4 μl stem-loop primer mix with concentrations set at 250 nM for target siRNA and reference miR-16. The 6 μl mix was incubated at 95°C for 5 min, 60°C for 5 min and then held at 4°C. After this pre-denaturation and annealing process, the resulting sample was then mixed with a 14 μl RT reaction mixture, comprising 0.25 μl RNAse inhibitor, 0.2 μl dNTPs, 1.3 μl MultiScribe^™^ reverse transcriptase, 2 μl RT buffer, and 10.25 μl nuclease-free water. The reaction mixture was incubated at 16°C for 30 min, followed by 42°C for 30 min, and a final inactivation step at 85°C for 5 min. The results with and without pre-denaturation and annealing process were compared.

The qPCR step was carried out using PowerUp^™^ SYBR^™^ Green Master Mix (Applied Biosystmes) in CFX96 RT-PCR Detection Systems (Bio-Rad Laboratories). A typical 20 μl qPCR reaction included 2 μl of RT product, 0.5 μl of 60 μM forward primer, 0.5 μl of 28 μM universal reverse primer, 10 μl of PowerUp^™^ SYBR^™^ Green Master Mix, and 7 μl nuclease-free water. The multiplex thermal reaction program was as follows: 2 min at 50°C, 2 min at 95°C, 40 cycles of 15 s at 95°C, and 1 min at 60°C. The forward primers and universal reverse primers for each target are summarized in Supplemental Table S2.

### Quantification of RISC-loaded siRNA amount

The quantification of RISC-loaded siRNA (antisense strand) involved immunoprecipitation (IP) [25] followed by the stem-loop RT-qPCR procedure described above. The standard curve was generated using the AS instead of the duplex siRNA.

Cell pellets with approximately 200,000 cells were lysed in 100 µl of IP lysis buffer (Pierce) containing RNaseOUT (1:200, Thermo Fisher) and a protease/phosphatase inhibitor cocktail (1:100, Pierce). The lysate was vortexed and incubated on ice for 30 min, followed by centrifugation at 20,000*g* for 10 min. The total protein concentration of the resulting supernatant (10 μl) was determined using a BCA kit (Pierce) for cell number normalization. An 85 μl aliquot of the supernatant was transferred to a new Eppendorf tube and incubated with Ago2 antibody (1.3 μg, ab57113, Abcam) while gently rotating at 4°C overnight. Subsequently, the lysates were mixed with Dynabeads Protein G magnetic beads (20 μl, Life Technologies) and rotated at 4°C for an additional 2 h. To release siRNA from Ago2, the magnetic beads were anchored to the side of the tube using a magnetic bar, washed three times with ice-cold PBS, incubated with 100 μl iScript SPR at 95°C for 5 min, and then centrifuged at 15,000*g* for 2 min at 4°C. The RISC-loaded siRNA in the supernatant was then quantified using the stem-loop RT-qPCR approach outlined above.

### Cell number normalization

Endogenous miR-16 was selected as the internal reference to normalize cell numbers for measuring cytoplasmic siRNA levels per cell. In different cell lines, 360,000 cells were accurately counted and lysed with 600 μl iScript SPR. The supernatant, representing 600 cells/μl, was then underwent three consecutive 2-fold dilutions using iScript SPR to obtain the following cell suspensions: 600, 300, 150, and 75 cells/μl. Cell number normalization standard curves were then established based on the miR-16 Ct values obtained from stem-loop RT-qPCR against known cell numbers.

The total protein concentration after cell IP lysis was utilized to normalize cell numbers during RISC-loaded siRNA quantification. Concentrations determined at various time points were used to calibrate the average total protein concentration per cell.

### mRNA determination

Total RNA was extracted using E.Z.N.A. HP Total RNA Kit (Omega Biotek), according to the manufacturer’s instructions and reverse transcribed to cDNA using iScript^™^ cDNA Synthesis Kit (Bio-Rad Laboratories). qPCR was performed with iTaq^™^ Universal SYBR^®^ Green Kit (Bio-Rad Laboratories) on CFX96 RT-PCR Detection Systems. The 18S ribosomal RNA served as the reference gene. The primer sets were purchased from IDT and their sequences are summarized in Supplemental Table S3. The multiplex thermal reaction program consisted of 3 min at 95°C, 40 cycles of 15 s at 95°C, 1 min at 60°C. Target mRNA levels at each time point, relative to the negative control siRNA-treated sample and the internal reference gene, were calculated using the ΔΔCt-method [26].

### Data analysis

The Ct values and normalized relative expression were obtained using CFX Maestro software (version 1.1, Bio-Rad Laboratories). GraphPad Prism (version 9.3, San Diego, CA, USA) was used to plot the data and perform the linear regression. PK parameters were calculated using Phoenix WinNonlin (version 8.3, Pharsight, Sunnyvale, CA, USA) from the mean time-profiles of cytoplasmic and RISC-loaded siRNA at different dosing levels.

## Results

### Reproducibility and linearity

**Figure 2.**
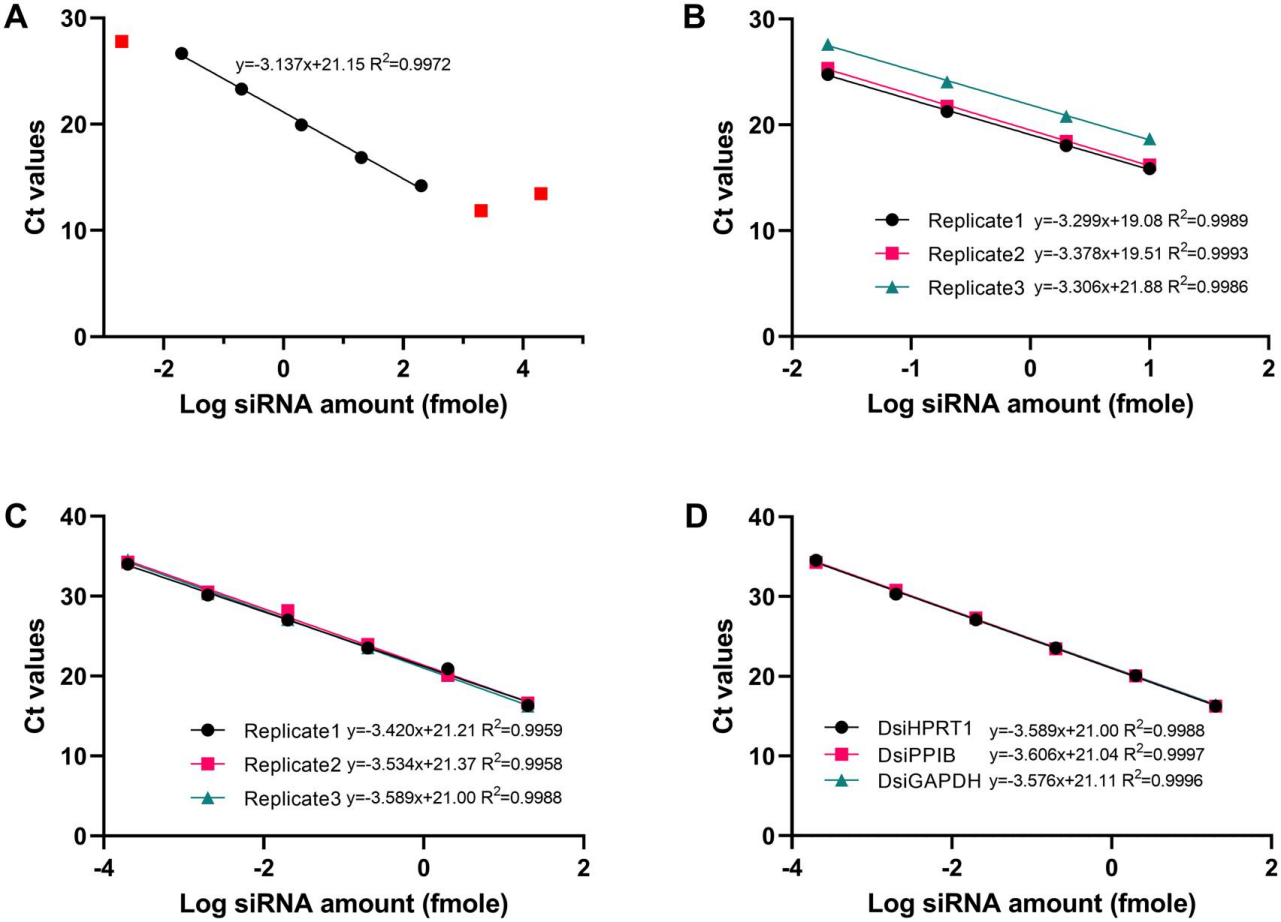
Comparison of calibration curves for different siRNA quantification methods. (A) Assessment of the linearity of the ploy(A)-based RT-qPCR method for DsiHPRT1 quantification. (B) Calibration curves of column-based siRNA extraction followed by poly(A)-based RT-qPCR method for DsiHPRT1 quantification. (C) Calibration curves of stem-loop RT-qPCR method DsiHPRT1 quantification. (D) Calibration curve comparison of stem-loop RT-qPCR method for different siRNAs. Each point represents the mean of triplicate determinations.

We initially assessed the suitability of chemical- and immobilization-based siRNA extraction coupled with a poly(A)-based RT-qPCR for DsiHPRT1 quantification. The sequences of DsiHPRT1 are listed in Supplemental Table S1. Spiked DsiHPRT1 samples (0.002 to 20,000 fmole) were extracted for poly(A) RT-qPCR. Ploy(A)-based RT exhibited nonlinearity beyond 200 fmole, affecting background signals below 0.002 fmole (Fig. 2A), defining a linear range of 0.02–200 fmole. For calibration, 10 fmole (10 µl of 1 nM stock solution) of siRNA was extracted, and the cDNA was appropriately diluted for the calibration curve. Although achieving good linearity (*R*^2^ > 0.99), poly(A)-based RT-qPCR exhibited poor reproducibility (Fig. 2B). Since siRNA extraction is the initial step for this assay, we evaluated the impact of the column extraction method on high variability. Using 200 fmole DsiHPRT1, we conducted the extraction ten times, followed by the same RT and qPCR reaction mix. We observed Ct value differences exceeding 2 (Supplemental Table S4), indicating significant assay variability in siRNA extraction alone [17, 27]. Additionally, the spiked internal standard (miR-16) was unable to correct this variation. We did not further assess the variation in subsequent steps. Even when considering the variation in siRNA extraction alone, this method may not be suitable for the absolute quantification of siRNA.

We then tested a simple cell lysis method without complex RNA purification for siRNA extraction. To mimic the actual cell lysis process, 10 µl of DsiHPRT1 stock solution was mixed with 100 µl of iScript SPR, followed by the stem-loop RT-qPCR system for siRNA quantification. The stem-loop RT primers and qPCR primers sets for different targets are summarized in Supplemental Table S2. This method demonstrated excellent reproducibility with desirable linearity (*R*^2^ > 0.99) from 0.0002 to 20 fmole (Fig. 2C). qPCR efficiency was acceptable (∼92%), based on the slopes of calibration curves. Interestingly, calibration curves for different siRNAs against different targets were highly identical (Fig. 2D). Hence, the simple cell lysis coupled with stem-loop RT-qPCR method was selected for further evaluation.

### Specificity

The specificity of stem-loop RT-qPCR mainly depends on the design of the stem-loop RT primer and the corresponding forward primer. In addition to the 6-bp overlapping stem-loop primer, we evaluated 9-bp and 10-bp overlapping primers against DsiHPRT1 and miR-16, respectively (Supplemental Table S5). Compared with the 6-bp overlapping primer against DsiHPRT1, the 9-bp overlapping primer showed no significant improvement, while exhibiting much higher background signals for blank samples and cell lysates without siRNA treatment (Supplemental Table S6). In addition, no significant difference was observed between the 6-bp and 10-bp overlapping primers for mi-R16. These results suggest that the 6-bp overlapping stem-loop primer is a more appropriate choice.

siRNA degradation appears to occur in a stepwise pattern, primarily from the 3′-end to the 5′-end of the strands [28]. To evaluate the specificity of the stem-loop RT-qPCR method for potential metabolites, we compared intact DsiHPRT1 (20 fmole) with DsiHPRT1 metabolites, each with one or two 3′-terminal nt removed (AS(N-1)3′, AS(N-2)3′) on the antisense strand (AS). This analysis revealed an average Ct delay of 2.8 and 8.1 for AS(N-1)3′ and AS(N-2)3′, respectively, compared to the full-length sequence. These results suggest that potential interference from metabolites in the quantification of intact siRNA is limited (<15%).

**Figure 3.**
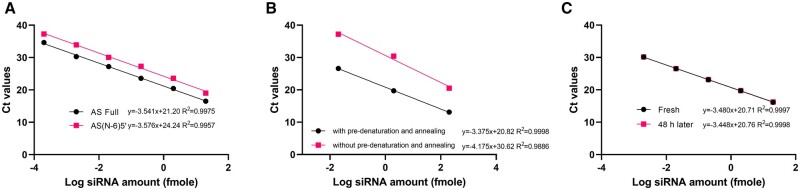
Assessment of factors affecting siRNA quantification. (A) Impact of removing the 6-nt sequence at the 5′-terminus of DsiHPRT1’s antisense strand on quantification. (B) Impact of pre-denaturation and annealing on stem-loop RT-qPCR quantification. (C) Stability evaluation of cDNA synthesized from DsiHPRT1 using stem-loop RT. Each point represents the mean of triplicate determinations.

Moreover, the 6-nt dicer-substrate sequence at the 5′-terminus of the DsiHPRT1 antisense strand will be eliminated during its RISC loading process [11]. In the quantification of total cytoplasmic siRNA amounts, RISC-loaded siRNA lacking the dicer substrate sequence can still be detected due to the shared 3′-terminus sequence. To assess the impact of removing the 6-nt sequence at the 5′-terminus of antisense strand, both DsiHPRT1 and DsiHPRT1 lacking the dicer substrate sequence were quantified utilizing the same stem-loop primer and forward primer. As shown in Fig. 3A, the 6-nt sequence removal at the 5′-terminus of the antisense strand resulted in an average Ct delay of 3.1 compared to the full antisense sequence. Considering the much lower RISC-loaded DsiHPRT1 amount, the stem-loop RT-qPCR method predominantly determines intact DsiHPRT1 in the cytosol.

### Pre-annealing of antisense strand to RT primer and the stability of cDNA

To evaluate the effect of siRNA duplex structure on the quantification of antisense strands, we conducted a comparison between samples subjected to the pre-denaturation and annealing process as described earlier and those without it. It turned out that the incorporation of pre-denaturation and annealing notably increased sensitivity by over two orders of magnitude (Fig. 3B). Moreover, this enhancement led to an improvement in linearity as well.

The stability of cDNA synthesized using the stem-loop primer is also a matter for siRNA quantification [29]. A portion of the freshly synthesized cDNA underwent immediate qPCR analysis, while the remaining portion was stored at 4°C for 48 h before being analyzed. The findings demonstrated that cDNA samples generated through the stem-loop primer-based RT exhibit remarkably stable at 4°C for at least 48 h (Fig. 3C).

### Impact of microcentrifuge tubes and cell numbers

**Figure 4.**
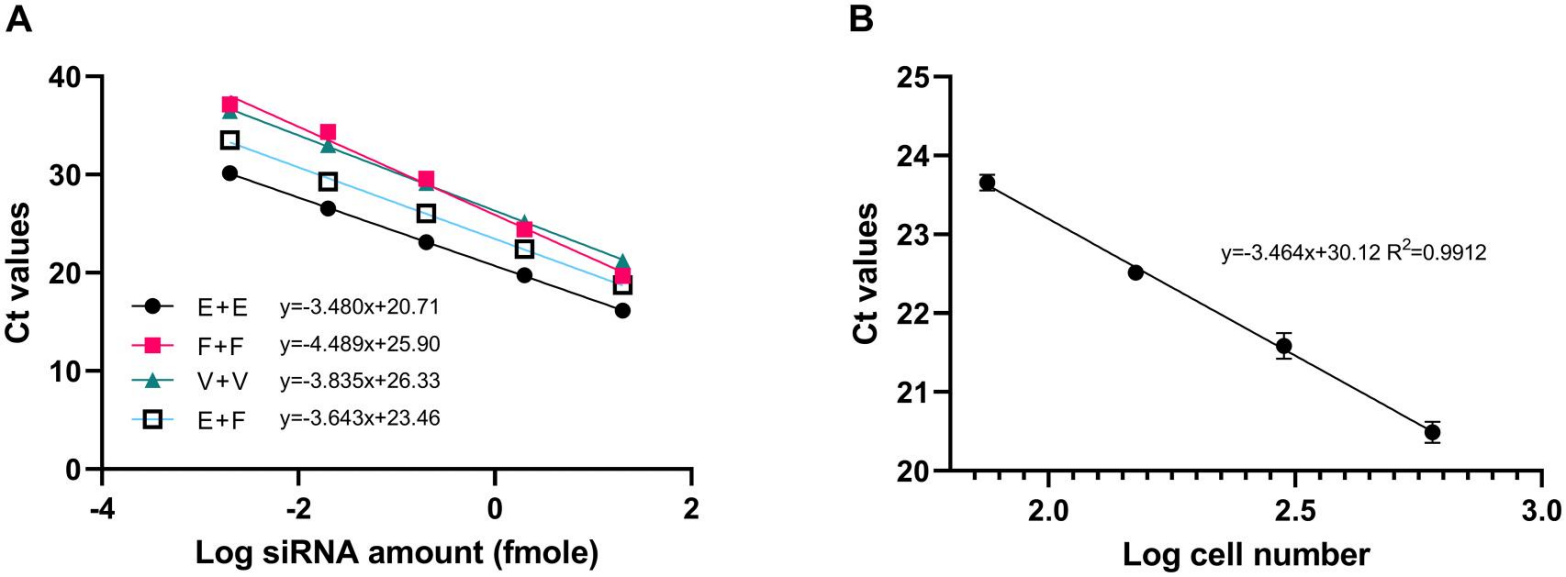
Assessment of microcentrifuge tubes and cell number normalization reference. (A) Impact of different microcentrifuge tubes on DsiHPRT1 sample storage and processing. E + E represents Eppendorf tube for storage and Eppendorf tube for processing; F + F represents Fisher tube for storage and Fisher tube for processing; V + V represents VWR tube for storage and VWR tube for processing; E + F represents Eppendorf tube for storage and Fisher tube for processing. (B) Calibration curve for BT474 cell numbers (75, 150, 300, and 600) based on endogenous miR-16. Each point represents the mean of triplicate determinations.

Given the exceptionally high sensitivity of our quantification method, even minor losses of siRNA during storage and sample processing can significantly impact results. To address this concern, we compared three different brands of microcentrifuge tubes: Eppendorf^™^ Snap-Cap Microcentrifuge Safe-Lock^™^ Tubes (05-402-98), VWR^®^ High Performance Microcentrifuge Tubes (20170-038), and Fisherbrand^™^ Low-Retention Microcentrifuge Tubes (02-681-320). All tubes were autoclaved for sterility during cell culture. Our findings indicated that tube selection indeed affected siRNA sensitivity. Eppendorf tubes, utilized for storage and sample processing, exhibited the highest sensitivity and primer efficiency >90% (Fig. 4A). Notably, this was the only combination to reach this level of efficiency. Moreover, a comparison among different tube groups revealed that tube adsorption occurred during both storage and processing steps.

Since our cell lysis method does not involve RNA isolation and purification, the presence of endogenous proteins and DNA can potentially interfere with subsequent RT-PCR. To determine the optimal cell numbers that can be processed with this reagent, we lysed precisely counted 1,000,000 BT474 cells using 200 μl iScript SPR. The supernatant was then used for four consecutive 5-fold dilutions, yielding cell suspensions of 5000, 1000, 200, 40, and 8 cells/μl. After mixing DsiHPRT1 (200 fmole, 10 μl of 20 nM) with 100 μl of each cell suspension, we quantified the siRNA and miR-16. We observed a noticeable Ct shift in the 5000 cells/μl lysate (Supplemental Table S7), while miR-16 exhibited acceptable linearity and primer efficiency from 5000 to 8 cells/μl. Consequently, we chose to use 20,000 cells lysed in 100 μl of iScript SPR (resulting in 200 cells/μl) for the quantification of DsiHPRT1. The cell number normalization standard curve was established from 600 to 75 cells/μl using miR-16 as the reference gene (Fig. 4B).

### Accuracy and precision

Intra- and inter-day accuracy and precision were assessed by determining four replicates of QC samples in three independent runs (Supplemental Table S8). The results demonstrate that the accuracy for all four levels of QC samples fell within ±15% of the nominal values. Additionally, the relative standard deviation values for both intra-day and inter-day precision remained below 10%.

### Intracellular pharmacokinetic and pharmacodynamic profiles of siRNAs

**Figure 5.**
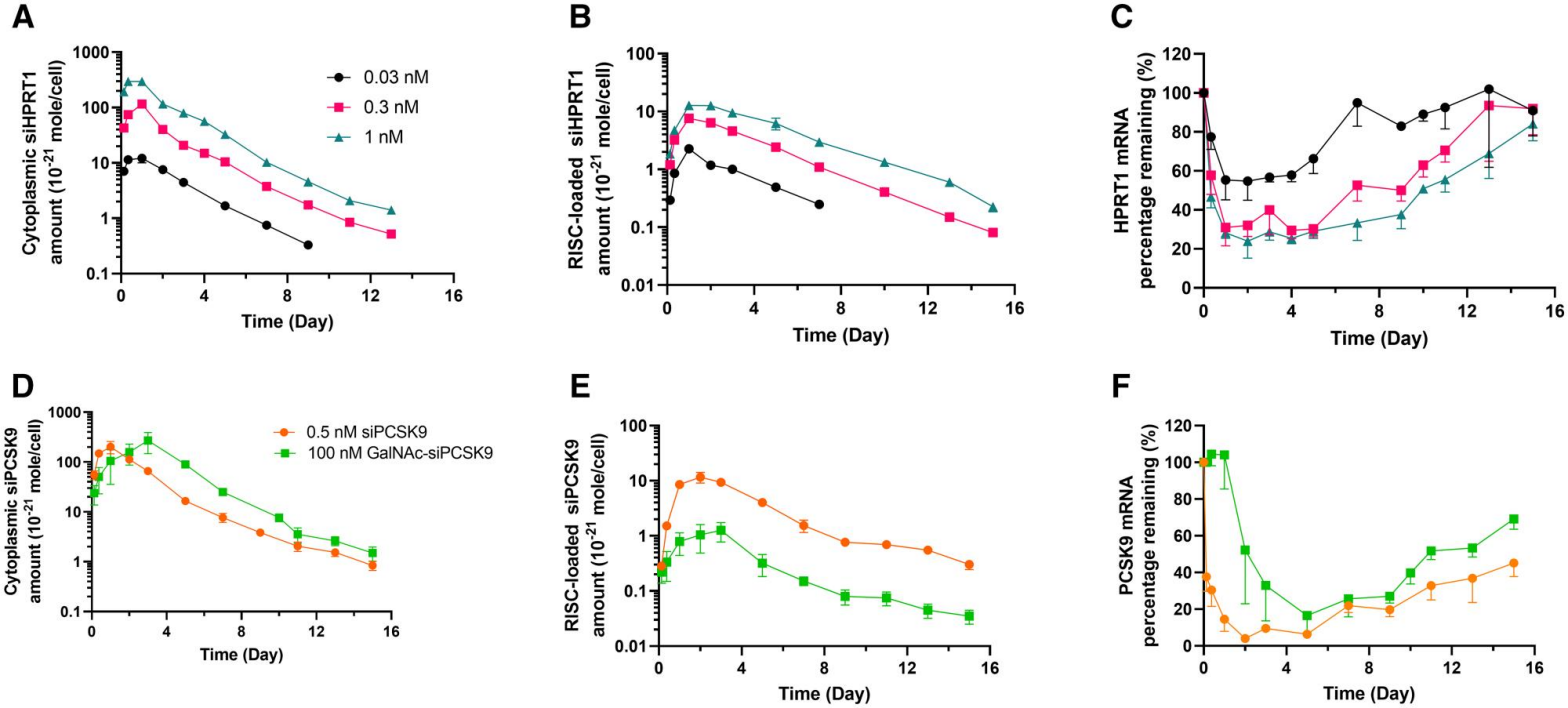
Time profiles of cytoplasmic siRNA, RISC-loaded siRNA, and mRNA knockdown. (A–C) siRNA and mRNA time profiles after 0.03, 0.3, and 1 nM of DsiHPRT1 treatment using RNAiMAX in BT474 cells for 24 h (*n* = 2 for siRNA PK and *n* = 3 for mRNA curves). (D–F) siRNA and mRNA time profiles of after siPCSK9 and GalNAc-siPCSK9 treatment in Hep3B cells (*n* = 4). Hep3B cells were incubated with Opti-MEM^™^ reduced serum medium containing 0.5 nM siPCSK9 (delivered in RNAiMAX) for 24 h or 100 nM GalNAc-siPCSK9 for 72 h. Each sample was determined in triplicate and data are represented as mean ± SD.

Our established stem-loop RT-qPCR method was successfully employed to investigate the intracellular disposition of siRNA, encompassing both cytoplasmic and RISC-loaded siRNA. Time profiles of cytoplasmic and RISC-loaded DsiHPRT1 in BT474 cells are shown in Fig. 5A and B, and the corresponding PK parameters are summarized in Table 1. Following a 24-h incubation with DsiHPRT1 at three dosing levels (0.03, 0.3, and 1 nM) in BT474 cells, the cytoplasmic DsiHPRT1 exposure (C_max_ and AUC) increased in a dose-proportional manner, indicating linear PK. In contrast, the exposure of RISC-loaded siRNA displayed a less than proportional increase at higher doses, indicative of nonlinear disposition. Terminal slopes and half-lives (approximately 2 days) remained consistent across doses. Notably, with escalating doses, the relative exposure of RISC-loaded DsiHPRT1 to cytoplasmic DsiHPRT1 decreased from ∼19% at 0.03 nM to ∼9% at 1 nM. In the 0.03 nM group, a 40% mRNA knockdown with 7-day recovery was observed, while the 1 nM group showed an 80% knockdown with over 16-day recovery (Fig. 5C). Through the comparison of extracted total RNA concentrations between experimental and control samples at each time point, we observed no significant cell loss after gene silencing over the prolonged culture period (data not shown).

**Table 1.** Pharmacokinetic parameters for cytoplasmic and RISC-loaded siRNA following the treatment with different siRNAs.

siRNA (Cell type)	Dose (nM)	Cytoplasmic siRNA PK			RISC-loaded siRNA PK			RISC/Cytoplasm C_max_ (%)	RISC/Cytoplasm AUC (%)
		*t*_1/2_ (day)	C_max_ (10^−21^ mole /cell)	AUC_0-inf_ (10^−21^ mole·day /cell)	*t*_1/2_ (day)	C_max_ (10^−21^ mole/cell)	AUC_0-inf_ (10^−21^ mole·day /cell)		
**DsiHPRT1 (BT474)**	**0.03**	1.71	12.1	35.6	1.99	2.27	6.78	18.7	19.1
	**0.3**	2.14	115.8	233.2	2.12	7.64	30.05	6.6	12.9
	**1**	2.09	296.6	728.3	2.23	12.72	65.01	4.3	8.9
**siPCSK9 (Hep3B)**	**0.5**	2.38	201.9	498.7	2.58	11.62	48.62	5.8	9.7
**GalNAc-siPCSK9 (Hep3B)**	**100**	1.97	269.2	894.5	2.52	1.26	5.04	0.47	0.56

t_1/2_: half-life; C_max_: siRNA maximum amount; AUC_0-inf_: area under the curve of siRNA amount versus time from 0 to infinity.

The developed method was also effectively applied to quantify chemically modified siPCSK9 and the GalNAc-siPCSK9 in Hep3B cells (Fig. 5D and E). The representative standard curve is displayed in Supplementary Fig. S1. When 0.5 nM siPCSK9 was delivered similarly to DsiHRPT1 using RNAiMAX (24-h treatment), its cytoplasmic exposure was approximately 30% lower than that of 1 nM DsiHPRT1, while the RISC exposure was similar. Treating Hep3B cells with 100 nM GalNAc-siPCSK9 for 72 h, where uptake occurs via GalNAc-ASGPR-mediated endocytosis, resulted in slightly higher cytoplasmic exposure compared to RNAiMAX-encapsulated 0.5 nM siPCSK9. Despite higher cytoplasmic concentrations, RISC-loading from GalNAc-siPCSK9 was slower and lower, with a RISC/cytoplasm exposure ratio of 0.5%, compared to 10% for RNAiMAX-delivered siPCSK9. Consequently, PCSK9 mRNA knockdown onset was delayed in GalNAc-siPCSK9-treated cells. Furthermore, RISC-loaded siRNA profiles well correlated with the extent of knockdown and recovery of PCSK9 mRNA compared to cytoplasmic siRNA profiles (Fig. 5F).

## Discussion

siRNA therapeutics have demonstrated substantial clinical benefits by targeting specific sequences to treat various diseases [4, 30]. Despite this potential, achieving a comprehensive quantitative understanding of siRNA intracellular disposition and trafficking, particularly its RISC loading process, is essential for its development and the establishment of a robust PK/PD relationship. This study introduces a convenient and sensitive stem-loop RT-qPCR method for quantifying cytoplasmic and RISC-loaded exposure of multiple siRNAs in *in vitro* cell model systems. The housekeeping gene HPRT1 in BT474 and the well-characterized gene PCSK9 in liver-related Hep3B were employed to evaluate the quantitative ability of proposed method. Additionally, the utilization of non-oncogenes in cancer cells can provide the preliminary understanding of the impact of rapid cell proliferation on intracellular siRNA PK/PD.

We conducted extensive evaluations, including different siRNA extraction methods, sample processing techniques, primer design, cDNA stability, microcentrifuge tube selection, and cell number normalization. While chemical extraction followed by immobilization is an effective method for siRNA extraction [17], our study revealed high variability (Fig. 2B), making it less suitable for absolute siRNA quantification. Moreover, this approach is both time-consuming and costly. In contrast, direct cell lysis for siRNA extraction presents a more efficient alternative. This method not only eliminates the complexity of RNA purification but also minimizes siRNA loss during processing, thereby enhancing data reproducibility (Fig. 2C). Additionally, we opted for the use of universal SYBR Green dye over specific TaqMan probes [16, 25] to improve the compatibility and cost–effectiveness of the stem-loop RT-qPCR method.

Despite the exceptionally high sensitivity of the RT-qPCR method, its ability to distinguish full-length AS from potential metabolites is still a concern. Our results indicate that the 6-bp overlapping stem-loop primer, coupled with a specific forward primer, can effectively distinguish intact AS from strands with even a single nucleotide missing at the 3′-terminus. This capability is crucial as AS degradation primarily occurs from the 3′-end, with AS(N-1)3′ being the major metabolite [28, 31]. Longer overlapping RT primer designs could compromise the ability to discriminate between intact AS and its metabolites [25]. Adjusting the primer set may also allow us to identify and quantify potential 3′-active metabolites. However, the stem-loop RT-qPCR method is less sensitive to 5′-terminal sequence loss [25]. For further characterization of 5′-metabolites, a mass spectrometry method with high specificity is a better choice [32].

While previous studies suggested potential instability of cDNA products derived from the stem-loop primer even at −80°C [29], our findings indicated minimal loss when these cDNA products were stored at 4°C for up to 2 days (Fig. 3C). This discrepancy may be attributed to storage and processing losses caused by microcentrifuge tube adsorption. Eppendorf^™^ Snap-Cap Microcentrifuge Safe-Lock^™^ Tubes were identified as the preferred choice, exhibiting minimal loss during both siRNA storage and processing (Fig. 4A). Although advanced Eppendorf^™^ DNA LoBind tubes were also evaluated, they did not demonstrate superiority.

To accurately determine the cytoplasmic siRNA amount in terms of per cell, reliable cell number correction is crucial. We selected miR-16 as a reference due to its abundant and consistent expression [23, 24]. Small error bars observed in cytoplasmic DsiHPRT1 and siPCSK9 profiles (Fig. 5A and D) confirm that miR-16 is an excellent choice for normalization. Although coimmunoprecipitation of miR-16 from RISC has been proposed for normalizing RISC-loaded siRNA quantification [23], our data showed decreasing RISC-loaded miR-16 levels with increasing RISC-loaded siRNA amounts (data not shown), suggesting potential competition for RISC loading between exogenous siRNA and endogenous miRNA [33, 34]. As an alternative, we employed total protein concentration after IP lysis for RISC-loaded siRNA normalization, maintaining a cell number for IP lysis close to ∼200,000 and calibrating total protein concentration per cell with multiple time points. It is important to note that the incubation of cell IP lysate and antibody should be conducted at a low temperature (e.g. 4°C) to prevent post-lysis loading of siRNA [23].

Our developed stem-loop RT-qPCR method has been effectively utilized to profile cytoplasmic and, notably, RISC-loaded amounts of chemically unmodified and modified siRNAs in different cell lines. The observed nonlinearity in the RISC loading of DsiHPRT1 suggests a potential saturation of this process (Fig. 5B). Additionally, the exposure differences between RISC-loaded siPCSK9 and GalNAc-siPCSK9 indicate distinct siRNA uptake and endosome escape mechanisms mediated by different delivery methods. Our future work aims to establish mechanistic mathematical models that delineate the siRNA uptake, endosomal escape, RISC-loading kinetics, and mRNA dynamics to quantitatively characterize the relationship among cytoplasmic siRNA, RISC-loaded siRNA, and concentration- and time-dependent mRNA knockdown. Furthermore, considering the significant influence of penicillin/streptomycin utilization in cell culture on gene expression and regulation [35], great caution should be exercised in the following studies focused on target identification, validation, and translation.

In summary, we developed a convenient and robust stem-loop RT-qPCR method with high sensitivity, enabling the investigation of intracellular disposition for both chemically unmodified and modified siRNA. Comprehensive assessments of key factors affecting siRNA quantitation, including siRNA extraction, sample processing techniques, primer design, microcentrifuge tube selection, and cell number normalization, were conducted. This method successfully characterizes time profiles of cytoplasmic and RISC-loaded siRNA, enhancing our understanding of its unique PK/PD properties.

## Authors’ contributions

Lin Chen (Conceptualization [lead], Formal analysis [lead], Investigation [lead], Methodology [lead], Project administration [equal], Writing—original draft [lead], Writing—review & editing [supporting]), Caroline Bosmajian (Conceptualization [supporting], Investigation [supporting], Methodology [supporting], Project administration [supporting], Writing—original draft [supporting]), and Sukyung Woo (Conceptualization [equal], Formal analysis [equal], Resources [lead], Supervision [lead], Writing—review & editing [lead]).

## Supplementary Material

bpae029_Supplementary_Data

## Date availability

The data underlying this article are available in the article and the online supplementary materials.
