# Supplementary material for: A highly sensitive stem-loop RT-qPCR method to study siRNA intracellular pharmacokinetics and pharmacodynamics
Source: Biol Methods Protoc. 2024 May 6;9(1):bpae029. doi: 10.1093/biomethods/bpae029 (PMC11112049; doi:10.1093/biomethods/bpae029)
Supplement: bpae029_Supplementary_Data [file bpae029_supplementary_data.pdf]

Supplementary Material

A Highly Sensitive Stem-loop RT-qPCR Method to Study siRNA Intracellular Pharmacokinetics and Pharmacodynamics

Lin Chen, Caroline Bosmajian, Sukyung Woo

Department of Pharmaceutical Sciences, School of Pharmacy and Pharmaceutical Sciences, University at Buffalo, State University of New York, Buffalo, New York 14214.

Corresponding Author:
Sukyung Woo, PhD
Associate Professor
Dept. Pharmaceutical Sciences
School of Pharmacy & Pharmaceutical Sciences
The State University of New York at Buffalo
352 Pharmacy Building
Buffalo, NY 14214
Phone: (716) 645-2466

Supplemental Table 1. Sequences for different siRNAs.

Targets	Sequence
DsiHPRT1	5’-GAG CUA UUG UAA UGA CCA GUC AAC A-3’ (sense)<br>5’-UGU UGA CUG GUC AUU ACA AUA GCU CUU-3’ (antisense)
DsiPPIB	5’-AGG AUU UGG CUA CAA AAA CAG CAA A-3’ (sense)<br>5’-UUU GCU GUU UUU GUA GCC AAA UCC UUU-3’ (antisense)
DsiGAPDH	5’-GCU CAU UUC CUG GUA UGA CAA CGA A-3’ (sense)<br>5’-UUC GUU GUC AUA CCA GGA AAU GAG CUU-3’ (antisense)
negative control siRNA	5’-CGU UAA UCG CGU AUA AUA CGC GUA T-3’ (sense)<br>5’-AUA CGC GUA UUA UAC GCG AUU AAC GAC-3’ (antisense)
miR-16	5’- UAG CAG CAC GUA AAU AUU GGC G-3’
siPCSK9	5’-CUA GAC CUG UTU UGC UUU UGU-3’ (sense)<br>5’-ACA AAA GCA AAA CAG GUC UAG AA-3’ (antisense)

Supplemental Table 2. Primers for stem-loop RT-qPCR analysis of different siRNA.

siRNA	Stem-loop RT primer	Forward primer	Universal reverse primer
**DsiHPRT1**	GTC GTA TCC AGT GCA GGG TCC GAG GTA TTC GCA CTG GAT ACG AC AAG AGC	GCGC TGT TGA CTG GTC ATT ACA ATA[1]<br>GCC GCC CTG GTC ATT ACA ATA[2]	CCA GTG CAG GGT CCG AGG TA
**DsiPPIB**	GTC GTA TCC AGT GCA GGG TCC GAG GTA TTC GCA CTG GAT ACG AC AAA GGA	GCGC TTT GCT GTT TTT GTA GCC AAA[1]<br>GCC CGC GTT TTT GTA GCC AAA[2]	
**DsiGAPDH**	GTC GTA TCC AGT GCA GGG TCC GAG GTA TTC GCA CTG GAT ACG AC AAG CTC	GCGC TTC GTT GTC ATA CCA GGA AAT[1]<br>GCG CGC GTC ATA CCA GGA AAT[2]	
**miR-16**	GTC GTA TCC AGT GCA GGG TCC GAG GTA TTC GCA CTG GAT ACG AC CGC CAA	GCGCCCG TAG CAG CAC GTA AAT A	
**siPCSK9/ GalNAc-siPCSK9**	GTC GTA TCC AGT GCA GGG TCC GAG GTA TTC GCA CTG GAT ACG AC TTC TAG	GCGC ACA AAA GCA AAA CAG GT	

[1]: for the quantification of cytoplasmic siRNA amount.
[2]: for the quantification of RISC-loaded siRNA amount.

Supplemental Table 3. Primers for RT-qPCR analysis of different mRNAs.

Target	Forward primer	Reverse Primer
HPRT1	CATTATGCTGAGGATTTGGAAAGG	CTTGAGCACACAGAGGGCTACA
18s	CTTAGAGGGACAAGTGGCG	ACGTGAGCCAGTCAGTGTA
PCSK9	AGGGGAGGACATCATTGGTG	CAGGTTGGGGGTCAGTACC

Supplemental Table 4. Ct values of DsiHPRT1 and miR-16 obtained through column-based siRNA extraction and poly(A)-based RT-qPCR.

Replicate	Ct value*	
	DsiHPRT1	miR-16
1	15.4	16.1
2	16.5	16.9
3	16.4	16.7
4	16.6	16.8
5	15.1	16.8
6	16.6	17.4
7	14.4	16.5
8	14.8	16.8
9	16.5	17.8
10	16.8	18.1

*Ct value represents the mean of triplicates.

Supplemental Table 5. Stem-loop RT primers and forward primers used to evaluate primer specificity for siRNA/miRNA determination.

Primers \ Target		DsiHPRT1	miR-16
6-bp overlapping assay	Stem-loop RT primer	GTCGTATCCAGTGCAGGGTCCGAGGTATTC GCACTGGATACGAC AAG AGC	GTCGTATCCAGTGCAGGGTCCGAGGTATTC GCACTGGATACGAC CGC CAA
	Forward primer	GCGC TGT TGA CTG GTC ATT ACA ATA	GCGCCCG TAG CAG CAC GTA AAT A
9-bp overlapping assay*	Stem-loop RT primer	GTCGTATCCAGTGCAGGGTCCGAGGTATTC GCACTGGATACGAC AAG AGC TAT	GTCGTATCCAGTGCAGGGTCCGAGGTATTC GCACTGGATACGAC CGC CAA TAT T
	Forward primer	GCGC TGT TGA CTG GTC ATT ACA	GCGCCCG TAG CAG CAC GTA

*10-bp overlapping for miR-16.

Supplemental Table 6. Comparison of different stem-loop RT primer designs.

Spiked sample	Ct Values	
	6-bp overlapping stem-loop primer	9-bp overlapping stem-loop primer[1]
**DsiHPRT1 (200 fmol)**	12.7	13.2
**DsiHPRT1 (10 fmol)**	17.4	17.6
**DsiHPRT1 (0.002 fmol)**	29.4	29.3
**Blank[2] (no siRNA)**	>39	31.0
**Cell lysate (untreated)**	38.6	29.8
**miR-16 (10 fmol)**	18.3	18.5

[1]10-bp overlapping primer for miR-16.
[2]iScript™ RT-qPCR sample preparation reagent.
Ct values represent the mean of triplicates.

Supplemental Table 7. Ct values of DsiHPRT1 and miR-16 in cell lysate containing different number of cells.

BT474 cell number	Ct value DsiHPRT1	Ct value miR-16
5000 cells/μL lysate	13.90	17.89
1000 cells/μL lysate	13.37	20.35
200 cells/μL lysate	13.15	22.82
40 cells/μL lysate	13.06	25.61
8 cells/μL lysate	12.94	27.74

Ct value represents the mean of triplicates.

Supplemental Table 8. Intra-day (n = 4) and inter-day (n = 12) accuracy and precision for stem-loop RT-qPCR quantification of DsiHPRT1.

**Spiked amount (fmole)**	**Intra-day**			**Inter-day**		
	**Measured amount (mean±sd, fmole)**	**Accuracy (%)**	**Precision (%)**	**Measured amount (mean±sd, fmole)**	**Accuracy (%)**	**Precision (%)**
**0.0005**	0.000519±0.0000379	103.8	7.3	0.000494±0.0000435	98.8	8.8
**0.008**	0.00813±0.000623	101.6	7.7	0.00812±0.000634	101.5	7.8
**0.5**	0.547±0.0216	109.4	3.9	0.532±0.0278	106.4	5.2
**16**	16.5±0.877	103.0	5.3	16.0±1.31	100.2	8.2

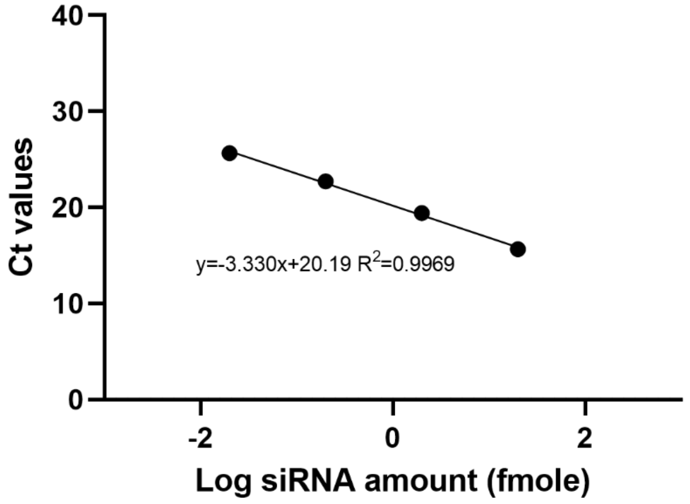

Supplemental Figure 1. The standard curve for chemically modified siPCSK9. Each point represents the mean of triplicate determinations.
